# Pathways to strengthening the epidemic intelligence workforce

**DOI:** 10.1186/s12919-025-00318-4

**Published:** 2025-02-28

**Authors:** Barbara Tornimbene, Zoila Beatriz Leiva Rioja, Olaolu Aderinola, Zulma M. Cucunubá, Catalina González-Uribe, Danil Mihailov, Steven Riley, Sang-woo Tak, Oliver Morgan

**Affiliations:** 1 World Health Organization Hub for Pandemic and Epidemic Intelligence, Berlin, Germany; 2 CPC Analytics, Berlin, Germany; 3 Nigeria Centre for Disease Control (NCDC), Abuja, Nigeria; 4 Pontificia Universidad Javierana, Bogotá, Colombia; 5 Data.Org, New York City, USA; 6 UK Health Security Agency (UKHSA), London, UK; 7 Korea Disease Control and Prevention Agency (KDCA), Cheongju, South Korea; 8 School of Medicine and Centre of Sustainable Development Goals for Latin America and the Caribbean, Universidad de los Andes, Bogotá, Colombia

**Keywords:** Epidemic intelligence, Public health surveillance, Data science capacity, Collaborative governance, Workforce development

## Abstract

The evolving landscape of public health surveillance demands a proficient and diverse workforce adept in data science and analysis. This report summarises discussions from the third session of the WHO Pandemic and Epidemic Intelligence Innovation Forum, focusing on workforce readiness and technological advancements in epidemic intelligence. The forum emphasizes the necessity of multidisciplinary surveillance teams equipped with advanced data skills. Digital tools play a transformative role in data collection and analysis, enabling real-time tracking, integration, and interpretation of diverse data sources. However, effective surveillance relies on inclusive representation and skill development. Collaborative surveillance and interdisciplinary training programs were emphasized as critical pathways to enhance workforce capacity, decision-making, and equity in public health. Case studies from Nigeria, Korea, the UK, and Colombia showcase the role of digital tools and contextual expertise in addressing surveillance gaps. Sustained institutional support, cross-sector partnerships, and investments in data literacy and workforce development are pivotal for creating resilient and inclusive public health systems.

## Introduction

The increasing complexity of public health challenges demands a surveillance workforce skilled in diverse fields such as epidemiology, informatics, governance, and data science. This workforce comprises trained professionals responsible for collecting, analysing, interpreting, and disseminating data to monitor and respond to public health events. Collaborative approaches between academia and government are also critical for designing surveillance systems that align with policy needs, and multifaceted skills are required to meet contemporary public health challenges, including critical thinking and the ability to interpret data for decision-making.

The demand for a multidisciplinary workforce has been driven by the need to address complex challenges such as those posed by Ebola virus disease (EVD) and COVID-19 [1–4]. Today, the workforce requires enhanced knowledge across fields including social, political, and data science, as well as representativeness of the communities they serve to ensure culturally relevant and effective decision-making. Studies on EVD and COVID-19 highlight how multidisciplinary expertise and contextual understanding were critical for timely and effective responses [1–4]). Furthermore, new digital tools, like “Tatafo” [5] in Nigeria, “Zoe” [6] in the UK, and AI and machine learning tools in Korea, are being developed to streamline information collection and analysis from diverse sources to navigate the complexities of modern data environments. Proficiency in utilizing these tools is a critical skill for the modern surveillance workforce. Finally, with the enhancement of automation the epidemic intelligence workforce will spend less time on data collection, preparation, and cleaning, and focus more on interpretation, contextualization, and communication of insights, requiring the multidisciplinary skills to translate data into actionable decisions and adapt to diverse public health contexts.

Building diverse data science capacity together with creating an inclusive workforce representation is also crucial for effective public health surveillance. Achieving inclusiveness involves considering factors such as gender, ethnicity, language, and socio-demographic factors. Investments in developing data science skills to maintain this representativeness is needed, and partnerships between academia and government can help develop relevant curricula.

During this session of the World Health Organization (WHO) Pandemic and Epidemic Intelligence Innovation Forum [7], held on 14 July 2022, the National Public Health Institutes of Korea [8], Nigeria [9], and the United Kingdom [10], as well as Pontificia Universidad Javeriana [11] Universidad de los Andes [12] from Colombia, and Data.org [13] discussed strengthening the epidemic intelligence workforce through new technological advances, investing in data science talent, and transmitting necessary skills. The meeting also emphasized the importance of governance and financial resources. The objective of this session was to explore the evolving landscape of public health surveillance demands for a multidisciplinary workforce skilled in data science, epidemiology, informatics, governance, and social sciences.

### Navigating technological advances in public health surveillance

The experience of national public health agencies and academia with regards to technological advances for public health surveillance tools in the past few years has highlighted the simultaneous evolution of professional competencies required for a contemporary workforce to successfully work with new digital solutions [14–17].

These are a number of examples of digital and data-related innovations occurring across public, private, and social sectors, and the broad recognition of the critical role data science will be playing for the epidemic intelligence workforce. The Nigeria Centre for Disease Control and Prevention (NCDC) has moved from a paper-based Integrated Disease Surveillance and Response (IDSR) [18] reporting method to using a nation-wide Surveillance Outbreak Response Management and Analysis System (SORMAS) [19], an open-source mobile and web application software platform designed as a digital business management system that supports the identification and monitoring of infection outbreaks and follow-up of cases and contacts, nation-wide. The NCDC has also launched *Tatafo—*the word Tatafo means “gossip” in Yoruba, a local language in Nigeria—a data mining tool that uses text mining, analysis, and natural language processing to retrieve information from over 1250 media sites to identify public health threats at an early stage [5], and they also curate a public health event management system called SITAware [20].

The Korea Disease Control and Prevention Agency (KDCA) managed to explore COVID-19 epidemiological links of confirmed cases and predict possible future infections, by triangulating data from various sources such as cellular phones, travel histories and credit card transactions [21, 22]. The agency is currently in the process of developing artificial intelligence and machine learning tools for event-based surveillance activities [23], as well as platforms for bio-surveillance [24, 25]and syndromic surveillance [24, 25]. Together with available resources for epidemic intelligence, like Epidemic Intelligence from Open Sources (EIOS) [26], they are trying to develop tools that better reflect the Korean context, for example by using their own social media platforms, and establishing a workforce that is more aware of the reality on the ground.

The TRACE-LAC [27]project, launched in 2022 by the Pontificia Universidad Javeriana and Universidad de Los Andes in Colombia, aims to address the limitations of public health surveillance identified during the COVID-19 pandemic in the region. TRACE [28] is an initiative by Data.org, and aims at driving learning opportunities and digital solutions to strengthen epidemic prevention and response efforts. TRACE focuses on three pillars: i. investing in data science for social impact talent by working together with local partners who have a record of accomplishment in enabling access to opportunity in the field, such as journalists; ii. facilitating collaborative action around TRACE open-source tools for epidemics analytics. iii. strengthening community engagement activities in the region and supporting partnerships to accelerate both the development of the technology and the learning opportunities. TRACE-LAC intends to build a high-quality open-source data toolkit for epidemics analytics, as well as an engaged user community to inform decision-makers in the response to epidemics in Latin America and the Caribbean. To do this, the project is partnering with Epiverse [28] in developing digital solutions, while also considering the socio-technical context in the development of these analytical tools, including civil society, stakeholders, companies, and decision makers. Specifically, TRACE-LAC is developing open-source R packages for basic analytics of routine epidemiological surveillance data, the design and analysis of population-based serosurveys, and vaccine coverage and effectiveness analysis.

In the UK, special studies, like the REACT project—one of the largest population surveillance studies in the world which measures the prevalence of SARS-CoV-2 in the general population in England—and the Office of National Statistics COVID-19 Infection Survey, were set up to respond to gaps in standard data streams identified by teams that used advanced analytics. This allowed for rapid deployment of large-scale real-time data collection during the pandemic that was accompanied with advanced automation for data management and data analytics. *Zoe* [6]*,* a COVID-tracking study app where users input their symptoms has been launched in 2020 and widely used in the United Kingdom during the most critical phases of the pandemic.

Technological advances in public health surveillance enhance data collection and integration capabilities, but their effectiveness relies on skilled professionals and governance structures to contextualize and apply data insights effectively [14–17].

### Understanding the epidemic intelligence workforce skillset

The experience from many countries, particularly during the COVID-19 pandemic, has highlighted how skills such as mathematical modelling, data science, programming, data visualization, and qualitative analysis—previously not widely associated with public health surveillance—have become essential for addressing complex public health challenges. Expanding the epidemic intelligence workforce skillset is now essential for the integration of data science with epidemiological methods. In countries like Nigeria, where local public health capacity is limited, including professionals with data science skills, such as software developers and statisticians, has helped automate many of the time-consuming tasks associated with data management, data preparation and cleaning, and data analysis and visualization.

Another key aspect of the surveillance workforce is interdisciplinarity. Public health surveillance activities are often carried out in coordination with other disciplines, for example to promote the intersection between human, animal, and environmental health, and a workforce trained in different disciplines can maintain, refine, and come up with innovative approaches. Digital tools can assist with data integration, but scientists must be equipped to address questions posed by decision-makers at all levels—policy, public health, community, and individuals—by drawing on evidence, insights from various disciplines, and the triangulation and translation of data.

Finally, data science has the potential to be used for social impact, but for it to be effective, digital tools must be developed with an understanding of the cultural context in which they are used. This requires a workforce that is representative of the communities that will use the data tools, and that is equipped with knowledge of data ethics and power dynamics in data collection and access, and can translate and communicate science to diverse decision-making audiences [14].

### Considerations for building new data science capacity

Participants at the meeting recognized the need to maintain and expand investment in the development of data science skills for the public health surveillance workforce and to find use cases for these skills in non-pandemic times. Building new data science capacity requires interdisciplinary training programs that integrate epidemiology, governance, and social sciences. Collaborative training initiatives, supported by academia and government partnerships, ensure that the workforce is equipped to address complex public health challenges.

According to Data.org, a non-profit organization that supports partnerships promoting data for social impact, there is potential to create three and a half million data jobs in social impact fields in low- and middle-income countries across the world over the next decade. Data.org establishes programs to deploy data science skills to solve some big societal problems, whether it is climate or pandemic prevention, or financial inclusion, or social justice. In 2021, they ran a global scan of the need for data science talent for social impact across the world, with a particular focus on low- and middle-income settings. They explored four pathways: how to get new talent, how to retrain existing talent, how to encourage transitional talent so people who work in data jobs in other sectors switch to social impact work, and finally how to improve the data literacy of decision makers. They found that there is a persistent workforce shortage in data science in sectors like public health, with equally persistent barriers that exclude women and people of colour from the field. During the COVID-19 pandemic some public health institutions around the world, faced with the fast-changing situation of the pandemic and in need of data entry capacity, resorted to hiring short-term data analysts and data scientists as consultants from the private sector at a huge cost to provide technical support to their cadre of field epidemiologists and surveillance officers. This model is not sustainable.

As electronic health records and IT tools for data management become more prevalent in low-resource settings, data science, informatics, and engineering skills are becoming essential also in non-pandemic time. Moreover, they present an opportunity for the workforce to develop the same skills on similar data structures, which are then vital during epidemics and pandemics. Building the next generation of diverse data talent for social impact is therefore essential to effectively address health challenges. With the right investments, training a diverse workforce has the potential to transform local economies in terms of data, technology, and innovation, and will strengthen public health capacity across the world.

### Meeting the needs of epidemic surveillance workforce in the future

Meeting future workforce needs requires integrating interdisciplinary approaches, fostering collaborative surveillance, and strengthening governance frameworks to ensure equity and sustainability in public health systems. During the session, different approaches were discussed for incorporating new skills into the public health surveillance workforce.

In general, much closer coordination is needed between academia and government for the development of curricula that include data science and data analysis components. The academic sector must have a clear understanding of the skills the public health surveillance workforce of the future needs and must promote a shift from theoretical teaching to applied skills, by allowing students to work with real data during their training to make sure they get the intangible expertise of working in a real environment. The format of capacity development programs should also be adapted to make the learning experience as manageable for surveillance professionals as possible. The combination of in-person training and online resources, keeping modules concise to fit their available time for learning, and the translation of materials to different languages will be essential.

Some institutions have opted for upskilling approaches to ensure their public health surveillance specialists are trained. Data.org set a target of training and helping get trained 1 million purpose driven data practitioners, working across sectors from public health and surveillance to climate to financial inclusion and to humanitarian aid. They are launching several hubs across the world where they are engaging both in the teaching of data science, for example with academic partners at master's level or bachelors’ level, but also with organisations who will consume that talent by building and maintaining digital public goods. They also fund fellowships and find opportunities for those students once they graduate to be employed in those areas. An example is the educational component of the TRACE-LAC project as part of the Epiverse program in Colombia. According to its training mandate TRACE-LAC will provide a free hybrid learning experience in Spanish, the most spoken language in the region. A series of short courses on Outbreak Analytics and Modelling will be implemented aimed at public health workers and field epidemiologists. Data.org also highlighted the importance of partnership engagement and the circular approach, where teaching hubs produce content, release it free and open-source across digital platforms, to be picked up by other partners to launch further hubs so that many more students can be trained.

Finally, it is not just about training individuals and getting them to work in a particular system, but also about the understanding that those individuals need to have an environment which is supportive of their work, and they need to be able to access a career pathway. Making attractive offers to professionals with these capabilities is one of the most challenging aspects for government agencies. Salaries, benefits, and career development opportunities in the public sector are still not competitive enough compared to what other sectors can offer. All participants agreed that an important step is ensuring that leaders understand the connection between data science and disease outbreak surveillance, and the key role the former plays in epidemic intelligence, to provide competitive and attractive positions in the public health job market. In this sense, decision-makers should also be part of the target audience of data literacy programs, because only by having a clear understanding of the value of this connection will they lead the deployment of resources needed to make it a reality. On their side, public health organizations will need to go back to government with clear strategies and working plans, and justifiable financial demands.

### Institutional conditions for the development of the workforce

A clear message from the discussion is that the strengthening of the public health surveillance workforce also depends to a large extent on support and alignment from institutional leadership [29]. The big push to attract and train data scientists for jobs in the public sector, private sector, and civil society must be complemented with efforts to create enabling institutional and leadership environments that place a high premium on the use of data and evidence. COVID 19 has shown that health and government systems' have weaknesses. And within this crisis, responding in terms of resource mobilization, equipment, trained personnel, and decision making proved to be very challenging.

Based on its experience during the pandemic, the United Kingdom's leadership decided to undergo institutional restructuring of its public health protection and infectious disease capability. The UK Health Security Agency (UKHSA) was established on 1st October 2021, and it provides policymakers with intellectual, scientific, and operational leadership at local, national, and global levels. To meet the need for more efficient divisions of tasks and the necessity of communicating science to a range of stakeholders, the agency has established the Data, Analytics and Surveillance Group, splitting it in three functional directorates: i. data operation; ii. analytics and data science; iii. and all-hazard intelligence. The data operations and the all-hazard intelligence directorates are in a sense a post pandemic addition as they support the work that in the past was considered the overall analytical output. In the case of Korea, on 12 September 2020, the status of the Korea Centers for Disease Control and Prevention (KCDC), a unit under the Ministry of Health and Welfare, was elevated to a Vice-Ministerial level and rebranded as the KDCA. Aside from strengthening its public health institutions mandate, the government also actively funds research and development for epidemic intelligence and its workforce and facilitates the talent shift form academia to industry, and vice versa, to make sure it is investing in promoting the next generation of data science workforce and science in general.

### Future outlook

The pandemic has raised awareness on the epidemic intelligence workforce competencies that need to be further supported, and it is undeniable that in the last decade technology has been key in the development and strengthening of public health surveillance activities. Strengthening epidemic intelligence requires a focus on multidisciplinary skills, collaborative governance, and sustained investments. By integrating diverse expertise and aligning surveillance systems with decision-making frameworks, nations can ensure equitable and effective public health responses.

One of the paths ahead will be to focus on expanding data science skills of the surveillance workforce. However, while technology opens an array of possibilities in terms of automation of processes, and access to real-time information, among other benefits, it is important to highlight that data systems must be fundamentally interrelated with social and technical factors. When thinking about how to increase the efficiency of the workforce, aside from the data science skillset, key knowledge is related to the human and cultural context, and the social effect of public health. Automated data systems can perform data collection, collation, and analysis, and help dissemination, but the human input remains essential in surveillance work, particularly when planning and designing surveillance systems, and while interpreting any obtained intelligence [30]. Experienced public health professionals must be able to draw informed conclusions from the data and apply them to the real world to solve problems and drive action.

This is not only important for a more effective division of tasks, but also to direct investments and support to approaches with better chances of success and sustainability. For example, machine-based information scanning capability requires a lot of computing resources and a lot of predeveloped solutions that can be very expensive. Many efforts have also been placed to finance technologies seeking to predict when and where the next pandemic will happen, but experts [31] have argued that a much simpler and more cost-effective way to mitigate outbreaks is proactive, real-time surveillance of human populations and the establishing of global networks of highly trained local researchers and public health responders, such as the WHO Global Outbreak Alert and Response Network (GOARN) [32].

Moreover, training opportunities for the epidemic intelligence workforce need to be accessible, tailored to specific social contexts, focused on interdisciplinarity and diversity. The investments that countries are making to establish a workforce versed in data science in conjunction with public health surveillance is quite a new drive. And it is gaining more, more and more momentum. Institutional support has clearly reinforced the mandates of the National Public Health Agencies presenting at the meeting, and it has contributed to strengthening the workforce capacity in the last years. However, since 2021, much of the innovations and capacity building has been focused on the response to the COVID-19 pandemic. Moving forward, countries will need to find effective ways to use a wide variety of information for public health surveillance, which will require a workforce that includes data science professionals. Moreover, ensuring that all countries are working to build their data science capacities for public health surveillance will be important not just to ensure a global network of contemporary surveillance systems, but also to ensure equity and culturally relevant solutions for all communities.

## Data Availability

Not applicable.

## Abbreviations

WHO: World Health Organization
KDCA: Korea Disease Control and Prevention Agency
NCDC: Nigeria Centre for Disease Control
TRACE: Training for Response and Analytics in Collaborative Epidemiology
EVD: Ebola Virus Disease
GOARN: Global Outbreak Alert and Response Network
SORMAS: Surveillance Outbreak Response Management and Analysis System
EIOS: Epidemic Intelligence from Open Sources
